# Integrative analysis identifies DCBLD2 and immune-related biomarkers for major depressive disorder: evidence from human peripheral blood, post-mortem brain, and rat models

**DOI:** 10.3389/fnhum.2026.1770103

**Published:** 2026-04-30

**Authors:** Xinyu Wu, Shaoming Zhou, Ping Yang, Jiayi Chen, Honghua Liu, Lu Shi, Xuehua Zhang

**Affiliations:** 1 School of Clinical Medicine, Hunan Brain Hospital, Hunan University of Chinese Medicine, Changsha, Hunan, China; 2 Department of Psychiatry, The Second People’s Hospital of Hunan Province, Changsha, Hunan, China

**Keywords:** DCBLD2, immunedys regulation, machine learning, major depressive disorder, prefrontal cortex

## Abstract

**Context:**

Major depressive disorder (MDD) is a prevalent mental illness, and inflammatory processes are considered a pivotal component of the pathogenesis of MDD. This study aims to identify novel biomarkers associated with the development of MDD and to elucidate the underlying immunological mechanisms.

**Methods:**

Mendelian randomization (MR) studies confirmed that two inflammatory cytokines are related to MDD. Subsequently, MDD-related transcriptomic datasets were retrieved from the Gene Expression Omnibus (GEO) database, and potential mechanisms were revealed by screening for differentially expressed genes (DEGs) and enrichment analyses. To identify key genes associated with MDD, we employed various machine learning methods, including univariate logistic regression, least absolute shrinkage and selection operator (LASSO) regression, and random forest algorithms, to construct a risk prediction nomogram and ROC curve to evaluate the diagnostic efficacy of candidate genes for MDD. Additionally, we analyzed the immune infiltration in MDD and healthy controls, investigated the expression of key genes in publicly available human post-mortem prefrontal cortex (PFC) transcriptomic datasets (bulk and single-cell) and validated candidate biomarkers in peripheral blood from an independent human cohort and in the PFC tissues of a chronic unpredictable stress (CUMS) rat model.

**Results:**

Subsequently, a six-gene diagnostic signature (DCBLD2, FZD5, GP1BA, MMP8, RNF144B, and SOCS1) was constructed using logistic regression, demonstrating good discriminative ability (AUC = 0.83). Immune infiltration revealed that the infiltration levels of seven types of immune cells were significantly different between the MDD and control groups, and were correlated with DCBLD2. Bulk RNA sequencing found that DCBLD2 was significantly overexpressed in the postmortem PFC of MDD. Single-cell RNA sequencing analysis of the MDD post-mortem PFC further localized DCBLD2 overexpression specifically within microglia and endothelial cells. Experimental validation indicates that six genes (DCBLD2, FZD5, GP1BA, MMP8, RNF144B, and SOCS1) exhibited significantly elevated expression in the peripheral blood of MDD patients. In the prefrontal cortex of rats subjected to the chronic unpredictable stress (CUMS) model, significant upregulation was only confirmed for the key gene DCBLD2.

**Conclusion:**

Immune dysregulation is closely related to MDD, and DCBLD2, FZD5, GP1BA, MMP8, RNF144B, and SOCS1 may represent candidate biomarkers and potential therapeutic targets for MDD, with DCBLD2 being a particularly promising candidate.

## 1 Introduction

In recent years, Major Depressive Disorder (MDD) has emerged as a common mental disorder, with its prevalence increasing annually. Approximately 300 million people worldwide are affected by MDD, making it one of the leading causes of disability (GBD 2017 Disease and Injury Incidence and Prevalence Collaborators, 2018). The World Health Organization (WHO) ranks MDD as the third highest disease burden, with projections indicating it will become the leading cause by 2030 (Bains and Abdijadid, 2025). Despite advancements in treatment modalities such as pharmacotherapy, psychotherapy, and physical therapy, approximately 30%–50% of patients exhibit treatment resistance or inadequate response due to the complexity of MDD’s etiology and the heterogeneity among individuals (Dudek et al., 2021). Clinical diagnosis and assessment of depressive disorders primarily rely on screening scales, interviews, and comprehensive evaluations of patients’ clinical symptoms, lacking objective biological markers (biomarkers) (Wiseman et al., 2025). Consequently, the diagnosis of depressive disorders heavily depends on clinicians’ experience and patient cooperation, which contributes to a substantial rate of misdiagnosis. Additionally, functional magnetic resonance imaging (fMRI), multimodal magnetic resonance imaging, positron emission tomography (PET), and electroencephalography (EEG) are frequently utilized as auxiliary methods for the clinical diagnosis of MDD (Zhang et al., 2023). However, these clinical examinations are costly, and their accuracy remains contentious. Therefore, identifying suitable biological markers for MDD continues to be a focal point of biological research.

Increasing evidence suggests that inflammatory responses play a critical role in the pathogenesis of MDD (Lang and Borgwardt, 2013). Clinical studies have found that patients with MDD exhibit an enhanced immune response to pathogens and stressors, characterized by elevated levels of various pro-inflammatory cytokines (such as interleukins, interferons, chemokines, and tumor necrosis factor). Notably, even during clinical remission, patients still show abnormalities in the levels of relevant cytokines and their soluble receptors in peripheral body fluids (Miller et al., 2009). Excessive or sustained inflammatory cytokine activity can disrupt various neuronal functions, including impaired neurotransmitter signaling, interruption of neurotransmitter synthesis, reuptake, and release. This consequently impairs the functionality of neural circuits (Blume et al., 2011). Therefore, there is a bidirectional relationship between cytokines and MDD (Beurel et al., 2020).

MDD is characterized not only by imbalances in the peripheral immune system but also by dysregulation of immune responses in the central nervous system, particularly in the prefrontal cortex (PFC). Activation of microglia has been observed in the PFC of MDD patients (Pantazatos et al., 2017). In the context of chronic stress and gender-specific depressive-like behaviors, the persistence of depressive-like behaviors in female mice is closely linked to abnormalities in TLR-4 signaling within microglia. This suggests that immune dysregulation in the prefrontal cortex may play a crucial role in the pathogenesis of MDD (Martín-Hernández et al., 2018).

Therefore, this study employed a multi-tiered integrative strategy: First, we used Mendelian Randomization (MR) to genetically corroborate the causal role of inflammatory dysregulation in MDD. Second, leveraging transcriptomic data, we aimed to identify peripheral blood immune-related biomarkers and construct a diagnostic model. Third, we sought to validate and prioritize the most promising candidates by examining their expression in publicly available human post-mortem prefrontal cortex datasets, a key brain region in MDD, at both bulk-tissue and single-cell resolution. Finally, independent clinical and animal model validations were performed to strengthen the biological relevance of our findings.

## 2 Materials and methods

### 2.1 Data sources and preprocessing

**Figure 1:**
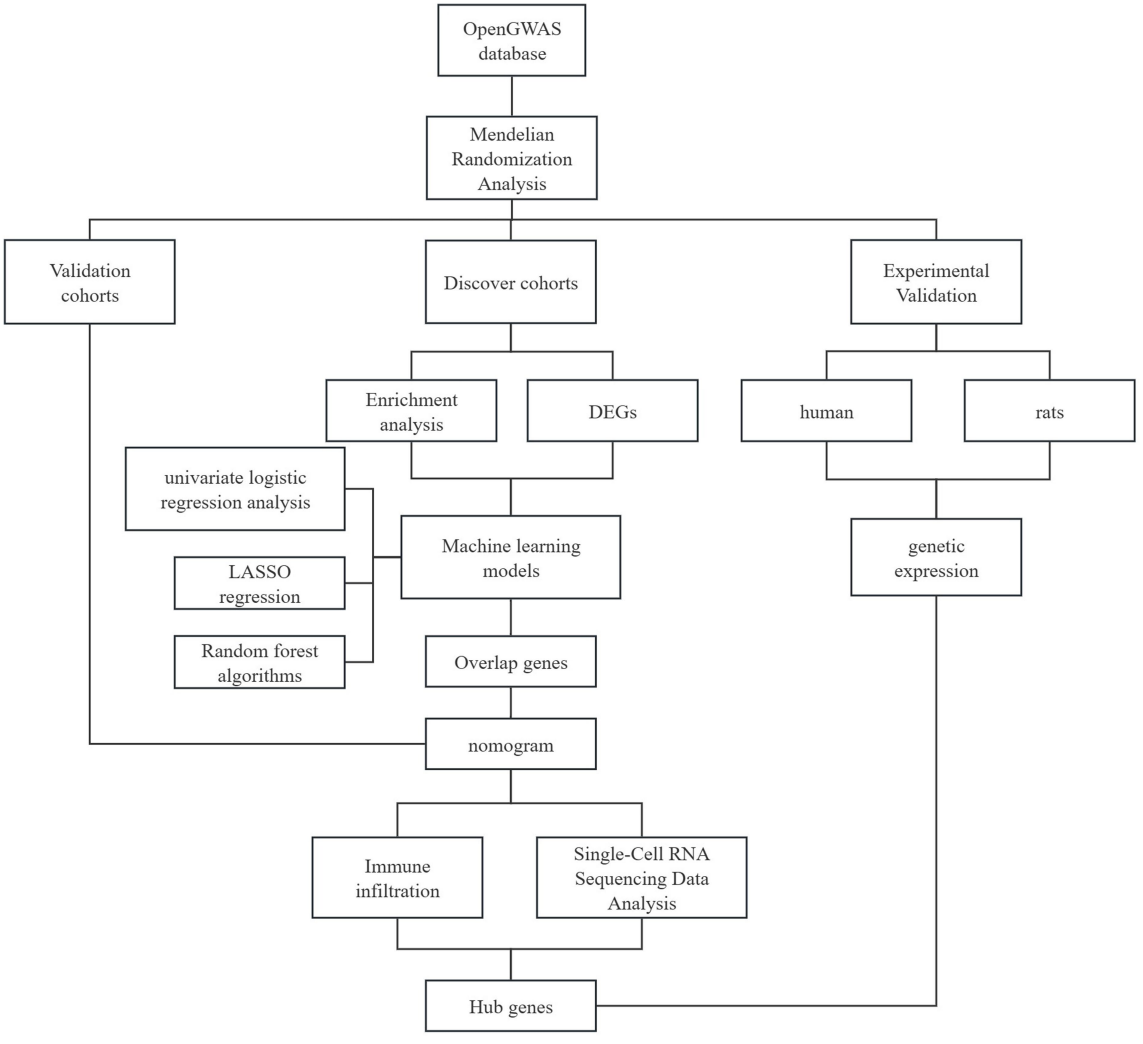
Schematic workflow of the integrative analysis for identifying and validating immune-related biomarkers in major depressive disorder (MDD).

Genome-wide association study (GWAS) summary statistics for Major Depressive Disorder (MDD) were obtained from the OpenGWAS database, comprising 135,458 cases and 344,901 controls. The study utilized the following datasets: GSE98793 (training set, whole blood), GSE247998 (validation set 1, peripheral blood), GSE38206 (validation set 2, PBMCs), GSE53987 (human post-mortem prefrontal cortex bulk RNA-seq), GSE144136 (human post-mortem prefrontal cortex scRNA-seq). All human brain tissue data were obtained from post-mortem samples, not from living patients. Genetic instruments for 41 inflammatory cytokines were sourced from a GWAS of 8,293 Finnish individuals (27989323). A schematic overview of the integrated analytical workflow, from data sourcing to experimental validation, is presented in Figure 1. Importantly, all human prefrontal cortex transcriptomic data used in this study (GSE53987 and GSE144136) were derived from post-mortem tissue samples, not from living individuals. This study did not involve the collection of new human brain tissue samples.

### 2.2 Mendelian randomization analysis

#### 2.2.1 Data sources

The GWAS data for 41 inflammatory cytokines originate from a study that provided genomic variation associations with 41 cytokines and growth factors in 8,293 Finnish individuals (27989323).

#### 2.2.2 Mendelian randomization analysis

For each inflammatory cytokine, single-nucleotide polymorphisms (SNPs) significantly associated (*p* < 1 × 10^−5^) were selected as instrumental variables. Linkage disequilibrium was clumped (*R*^2^ > 0.1 and within 500 kb) to ensure independence. A relaxed significance threshold (*p* < 1 × 10^−5^) was adopted to secure a sufficient number of instrumental variables for cytokines, given the moderate sample size of the source GWAS. To ensure instrument strength and mitigate weak instrument bias, we calculated the *F*-statistic and retained only SNPs with *F* > 10 for the final analysis. The primary MR analysis was performed using the inverse-variance weighted (IVW) method with random effects. Sensitivity analyses were conducted using MR-Egger, weighted median, simple mode, and weighted mode. Heterogeneity was assessed using the IVW *Q*-test and MR-Egger regression, while horizontal pleiotropy was evaluated using MR-Egger intercept test and MR-PRESSO global test. A leave-one-out analysis was performed to determine if results were driven by any single influential SNP (Richmond and Davey Smith, 2022).

### 2.3 Identification of diagnostic biomarkers and model construction

Differential gene expression analysis between MDD and control samples in the GSE98793 dataset was performed using The R package limma (v3.58.1) (Ritchie et al., 2015), the screening criteria are: ||log2FC| > mean(log2FC) + 2SD, *p*-value<0.05, a data-driven thresholding method recommended for robust identification of outliers in gene expression distributions (Wang et al., 2024). Functional enrichment analysis of DEGs for Gene Ontology (GO), Kyoto Encyclopedia of Genes and Genomes (KEGG), and Hallmark gene sets was conducted using the R package clusterProfiler (Kanehisa et al., 2023). To identify diagnostic biomarkers, inflammation-related DEGs were first screened by univariate logistic regression (*p* < 0.05). Subsequently, the least absolute shrinkage and selection operator (Lasso) and Random Forest algorithms were applied for further feature selection. The final diagnostic model was constructed using multivariable logistic regression, and a risk score was calculated based on the gene expression levels and their regression coefficients. A nomogram was developed to visualize the model. The model’s performance was evaluated using calibration curves and decision curve analysis (DCA).


$$\begin{array}{l} P(\text{target}=1)\\ =\frac{1}{1+\exp(-(\begin{array}{l} -47.86+2.72*\text{DCBLD}2+2*\mathrm{FZD}5+0.76*\mathrm{GP}1\mathrm{BA}\\ +0.68*\mathrm{MMP}8+1.1*\mathrm{RNF}144B+2.01*\text{SOCS}1 \end{array}))} \end{array}$$

### 2.4 Immune infiltration analysis

Immune cell infiltration was estimated using the CIBERSORT (R script v1.03) algorithm (Zhou et al., 2023). To corroborate the immune infiltration findings, two additional algorithms were employed on the training set (GSE98793): (1) The ESTIMATE algorithm was used to calculate stromal, immune, and estimate scores. (2) Single-sample Gene Set Enrichment Analysis (ssGSEA) was performed to quantify the enrichment scores of 28 immune cell types using gene signatures.

### 2.5 Single-cell RNA sequencing data analysis

Single-cell data were analyzed using Seurat v4.1.1, filtering out cells with mitochondrial content exceeding 20%, hemoglobin content exceeding 5%, or gene expression of fewer than 200 or more than 8,000 genes. This threshold, commonly used for complex neural tissue datasets where certain cell types exhibit high metabolic activity, was chosen to balance the removal of low-quality cells while preserving biologically relevant populations. Data normalization, cell clustering, and dimensionality reduction were performed using the Seurat package. The Find Variable Features function was used to select 2000 highly variable genes from the corrected expression matrix, followed by principal component analysis (PCA) using the RunPCA function, retaining the top 20 principal components for further analysis. Batch effects were corrected using the RunHarmony function from the R package harmony. Cell clustering was performed with the FindClusters function (resolution 0.6), and non-linear dimensionality reduction was conducted using the RunUMAP function. Cell clustering was annotated based on the CellMarker database and manually collected cell-specific markers.

### 2.6 Experimental validation

All human and animal studies were approved by the Ethics Committee of Hunan Second People’s Hospital (Approval No: 2022045). All human participants or their legal representatives provided written informed consent. Patients with MDD were recruited from the inpatients of Hunan Second People’s Hospital. Healthy controls were recruited from the local community. (Detailed inclusion and exclusion criteria are provided in the Supplementary Information). Sixteen male Sprague–Dawley (SD) rats (8–9 weeks old, weighing 200–220 g) were purchased from Spefu (Beijing) Biotechnology Co., LTD., with the production batch number N0110324231100438102, the Animal Experiment Center of Hunan University of Chinese Medicine. All animals were housed under a 12/12 h light/dark cycle in a controlled environment (temperature: 22 ± 2 °C, humidity: 55 ± 10%) with ad libitum access to food and water. Eight-week-old male Sprague–Dawley rats (*n* = 16) were randomly divided into a control group (*n* = 8) and a chronic unpredictable mild stress (CUMS) group (*n* = 8). The CUMS procedure lasted for 28 days and involved various stressors applied randomly (e.g., cage tilt, water/food deprivation, continuous illumination, restraint, wet bedding, mild foot shock, cold swim, tail clip). All efforts were made to minimize animal suffering. Based on the methods in the references and literature (Lin et al., 2021), the open field test was appropriately modified to assess depressive-like behaviors: an opaque material was used to make an open field experimental device with a size of 100 cm × 100 cm × 50 cm, and the bottom surface was evenly divided into 25 equal-sized squares. The rats were placed in the central square, and then the number of squares moved by the rats within 5 min (horizontal movement times) and the number of times the rats stood on their front limbs (vertical movement times) were observed. At the end of the experiments, rats were deeply anesthetized via intraperitoneal injection of tribromoethanol (avertin; TIGERGENE) at a dose of 1.5 mL per rat. After confirming the loss of reflexes, euthanasia was performed by cervical dislocation. Death was verified prior to tissue collection. Total RNA was extracted from human PBMCs and rat prefrontal cortex tissues. Reverse transcription was performed using the Evo M-MLV kit. Quantitative PCR was conducted using PerfectStart® Green qPCR SuperMix on an ABI QuantStudio 1 instrument.

For experimental validation, we focused on the six candidate genes identified by our diagnostic model (DCBLD2, FZD5, GP1BA, MMP8, RNF144B, and SOCS1). In human peripheral blood samples, the expression of all six genes was examined. In rat prefrontal cortex tissues, we selected three genes based on their expression patterns in the dataset (GSE53987) and functional relevance: DCBLD2 (a core gene significantly upregulated in MDD), FZD5 (a Wnt pathway receptor involved in neuroplasticity), and RNF144B (an E3 ubiquitin ligase regulating protein homeostasis and apoptosis). The primer sequences for rat DCBLD2, FZD5, RNF144B, and ACTIN are listed in Table 1; the primer sequences for human DCBLD2, FZD5, GP1BA, MMP8, RNF144B, SOCS1, and ACTIN are listed in Table 2.

**Table 1:** Rat DCBLD2 F and Rat DCBLD2 R primer sequences.

Primer name	Sequences (5′to 3′)
Rat actin F	CGTTGACATCCGTAAAGACCTC
Rat actin R	TAGGAGCCAGGGCAGTAATCT
Rat DCBLD2 F	GACCAAAGATGTTGCACTGGC
Rat DCBLD2R	CCTCGTGTTCCACCGATTTG
Rat FZD5 F	TCTCGACGGGGCCATAAAAG
Rat FZD5 R	CACTTGCTTGTGGTATGCGG
Rat RNF144B F	GGCTCAGTCCTCAAGGGATA
Rat RNF144B R:	AGGCAGAGCTTGCAAGTGAC

**Table 2:** Human DCBLD2 F and human DCBLD2 R primer sequences.

Primer name	Sequences (5′to 3′)
Human actin F	TCCTTCCTGGGCATGGAGT
Human actin R	AGCACTGTGTTGGCGTACAG
Human DCBLD2 F	TTGGTGGAAAGGAATGAAGC
Human DCBLD2 R	CTGCAGCACTGTGGTGACTT
Human SOCS1 F	CCTTAGCGTGAAGATGGCCT
Huamn SOCS1 R	CGAAGAGGCAGTCGAAGCTC
Human RNF144B F	CCCATCACTTGCCCTGACAT
Human RNF144B R	TGTTCGGTAGGGGTCCAGAT
Human MMP8 F	CTCCCTGAAGACGCTTCCAT
Human MMP8 R	TCCAGGTAGTCCTGAACAGT
Human GP1BA F	GGACACTGAGGGCGATAAGG
Human GP1BA R	AGGGGGTTGTATGGGCTTTG
Human FZD5 F	ACACCCGCTCTACAACAAGG
Human FZD5 R	CACTGAAGGACGGCTGGTAG

### 2.7 Statistical analysis

All experimental data were analyzed statistically using SPSS 27 64-bit software. The Shapiro–Wilk test was used to assess the normality of the data. Continuous variables are expressed as mean ± standard error of the mean (mean ± SEM) or median (interquartile range). Categorical variables are presented as *n* (%). In behavioral tests and gene expression validation (qPCR), comparisons between the control group and the CUMS model group were performed using two-tailed independent samples *t*-tests. The significance level for all statistical tests was set at *p* < 0.05. Statistical significance is indicated by asterisks, with the following standards: *p* < 0.05 (*), *p* < 0.01 (**), *p* < 0.001 (***), *p* < 0.0001 (****).

## 3 Result

### 3.1 Mendelian randomization analysis

**Figure 2:**
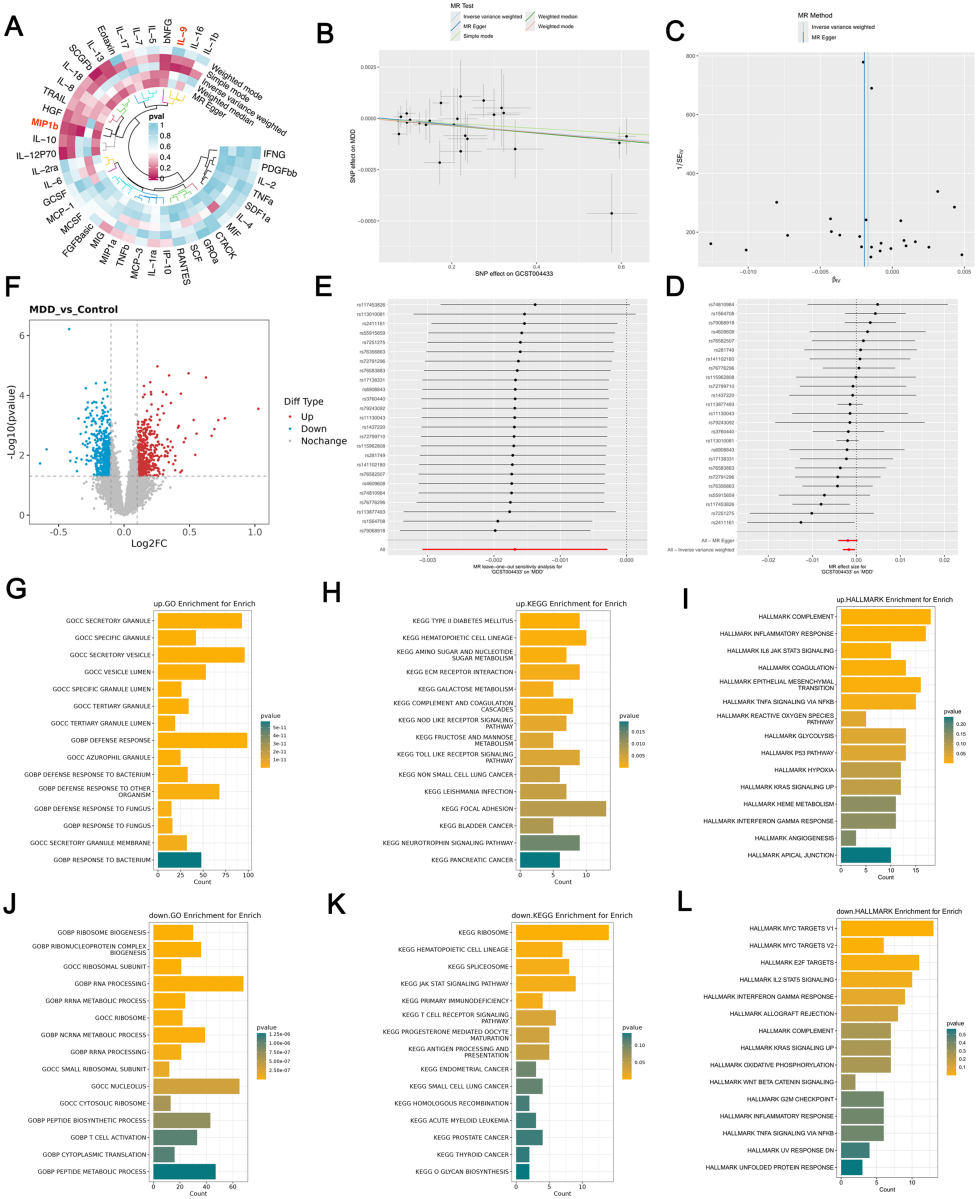
Mendelian randomization and functional enrichment analysis. **(A)** Heatmap of the causal associations between inflammatory factors and MDD. **(B–E)** Sensitivity analyses of the MR results for MIP-1β using IVW, MR-Egger, MR-PRESSO, and leave-one-out methods. **(F)** Volcano plot of DEGs between normal control samples and MDD samples in the GSE98793 dataset, where red and blue nodes represent upregulated and downregulated DEGs, respectively. **(G–I)** GO, KEGG, and Hallmark enrichment analysis of upregulated DEGs. **(J–L)** GO, KEGG, and Hallmark enrichment analysis of downregulated DEGs.

The MR analysis identified several potential associations, among which MIP-1*β* and IL-9 showed the most significant and robust causal relationships with MDD after sensitivity analyses (Figure 2A). The results indicate that elevated levels of M1P1b can reduce the risk of MDD, and this finding has been validated through various sensitivity analyses, demonstrating robustness. The MR Test shows consistent trends across different computational methods. A slope less than 0 indicates that the exposure factor is a favorable factor for the outcome, meaning that an increase in GCST004433 (MIP1b) corresponds to a reduced risk of developing MDD (Figure 2B). The segments calculated by the MR Egger and IVW methods for multiple SNPs are entirely positioned to the left of 0, indicating that an increase in GCST004433 (MIP1b) can lower the risk of MDD (Figure 2D). In the leave-one-out plot, each entry represents the MR analysis results of the SNP subset after excluding that SNP, with the corresponding x-axis representing the effect size estimates (i.e., the effect size estimates after leave-one-out analysis). As shown in the results, removing any SNP does not fundamentally affect the results (all lines are positioned to the right of 0), indicating that this MR result is indeed robust (Figure 2E). The funnel plot demonstrates that the MR Method segments are approximately symmetrical on both sides, indicating no heterogeneity (Figure 2C).

### 3.2 Differential gene analysis

#### 3.2.1 Functional enrichment analysis of upregulated genes

Differential gene analysis was conducted on the MDD samples and control samples from the GSE98793 dataset, resulting in a total of 1,188 differentially expressed genes (DEGs), of which 532 were upregulated and 656 were downregulated (Figure 2F). Functional enrichment analysis of the upregulated genes revealed significant enrichment in GO terms such as defense response and DEFENSE RESPONSE TO OTHER ORGANISM, which are associated with the organism’s defense mechanisms against pathogens or other foreign invasions, including the activation and execution of immune responses (Figure 2G). The KEGG enrichment results also highlighted immune response-related pathways: the Toll Like Receptor Signaling Pathway (crucial for innate immune responses mediated by Toll-like receptors), Complement and Coagulation Cascades (pathways involved in blood coagulation and complement system activation, essential for inflammatory responses and immune defense), and the NOD-Like Receptor Signaling Pathway (involved in NOD-like receptor-mediated inflammatory responses and cell death signaling) (Figure 2H). The Hallmark enrichment results indicate that the differentially upregulated genes in MDD samples are associated with inflammatory responses (Figure 2I): Complement (the complement pathway, which is part of the immune system, involves a cascade of proteins crucial for pathogen clearance, promoting inflammation, and regulating immune responses). Inflammatory Response (describes the response of cells and tissues to injury or infection, including vasodilation, leukocyte recruitment, and cytokine production). IL6 JAK STAT3 Signaling (describes how interleukin 6 (IL-6) signals through the JAK–STAT pathway, influencing cellular inflammatory responses and proliferation).

#### 3.2.2 Functional enrichment analysis of downregulated genes

Functional enrichment analysis of the 656 down-regulated DEGs revealed a distinct pattern complementary to the up-regulated genes. GO and KEGG analyses showed significant enrichment in terms related to ribosome biogenesis, RNA processing, and metabolic pathways (Figures 2J,K), suggesting a broad suppression of basic cellular functions. Importantly, down-regulated genes were also enriched in key immune-regulatory pathways such as the JAK–STAT signaling pathway and T cell receptor signaling pathway (Figure 2L). This indicates that the immune dysregulation in MDD may involve not only innate immune activation but also a concomitant impairment of adaptive immune and cytokine signaling functions.

### 3.3 Key risk genes and model construction

**Figure 3:**
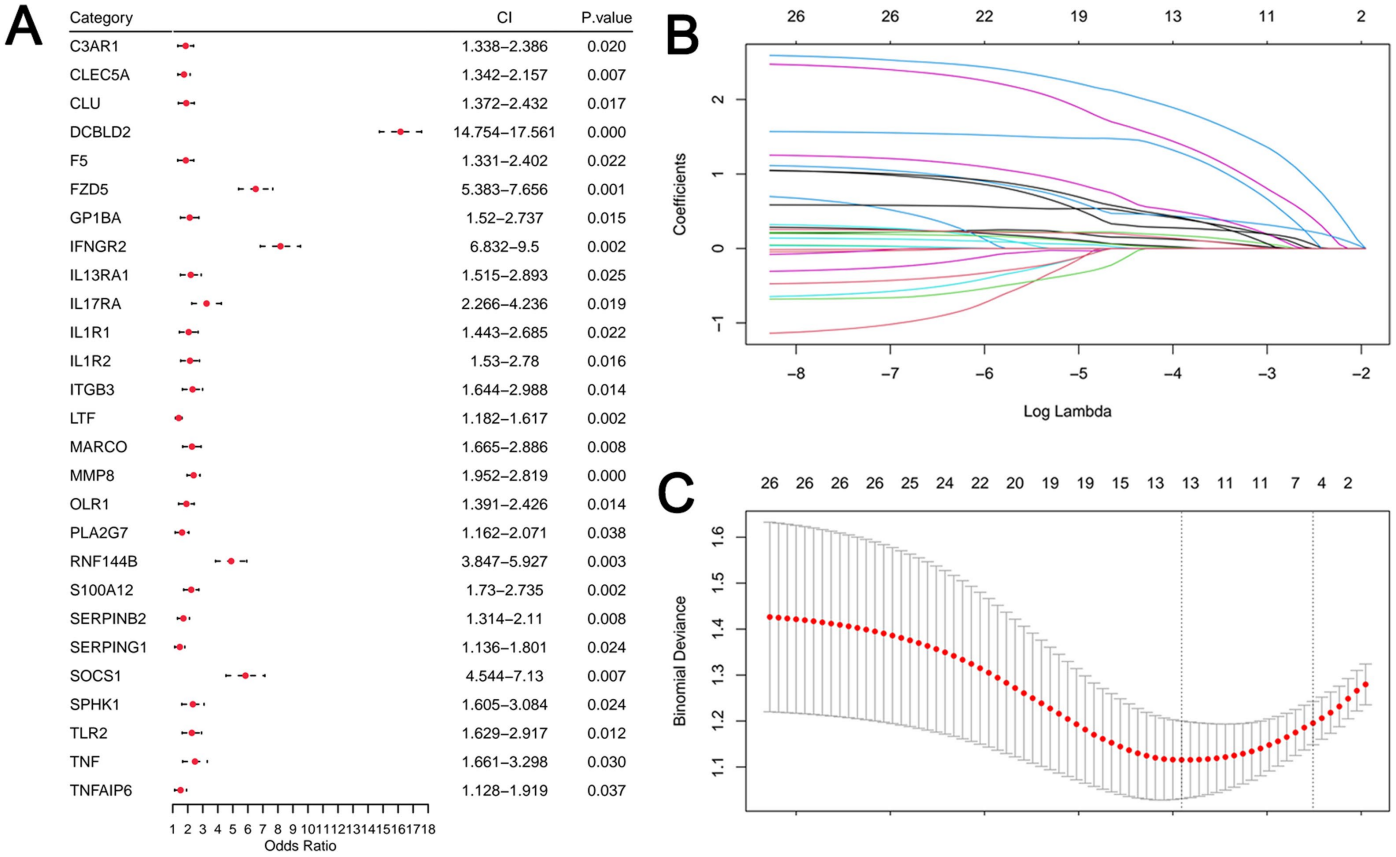
Analysis of MDD risk-related gene screening **(A)** Univariate logistic regression analysis identified a total of 27 genes associated with MDD risk (*p* < 0.05). **(B, C)** Coefficient path diagram and cross-validation error plot from LASSO regression analysis.

**Figure 4:**
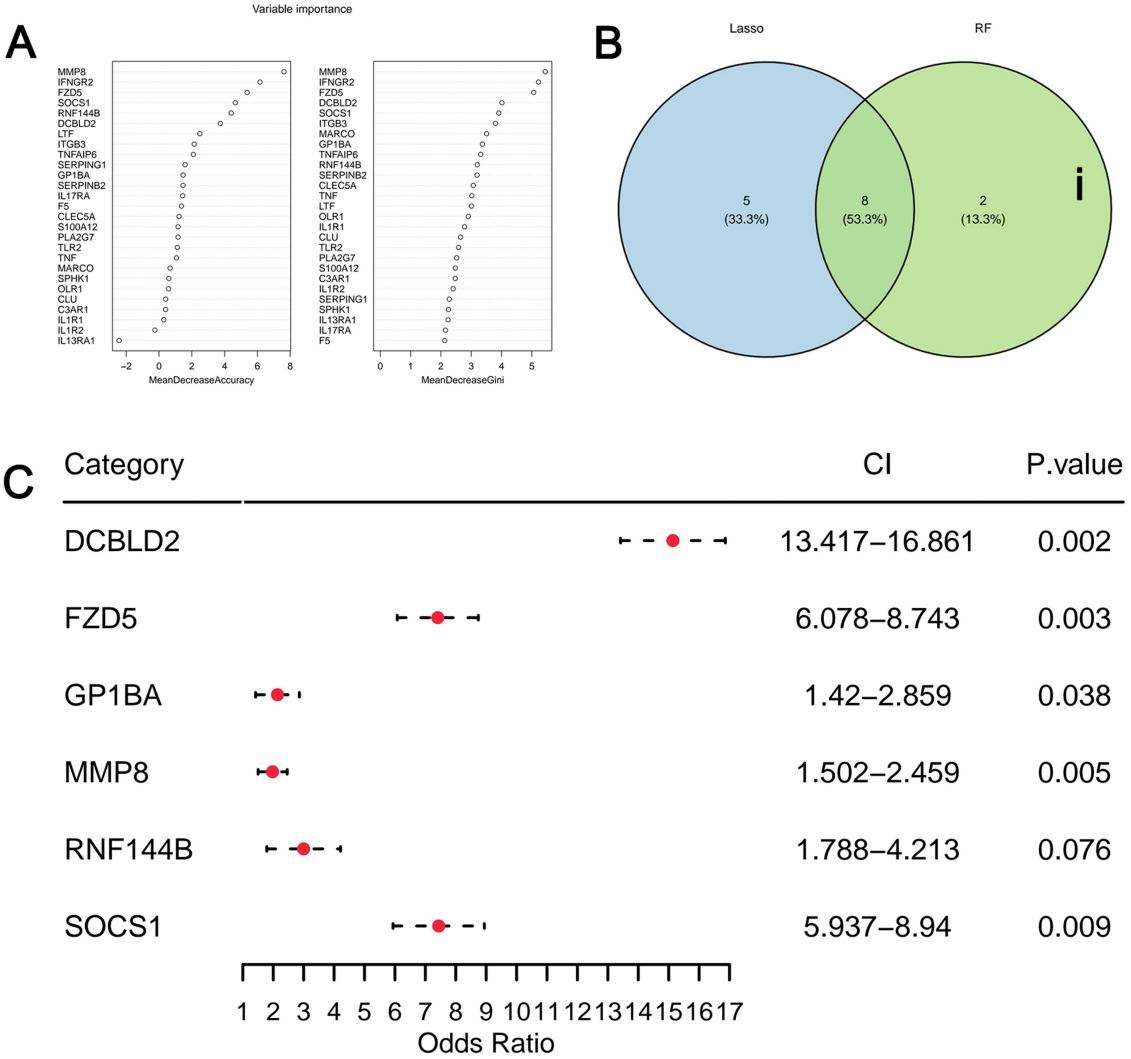
Six diagnostic biomarkers for model construction **(A)** Random forest model. **(B)** Venn diagram showing the intersection of feature genes from LASSO regression analysis and random forest algorithm, resulting in 13 genes related to MDD. **(C)** Multivariate regression analysis of six diagnostic biomarkers for model construction. Statistical significance is indicated by asterisks, with the following standards: *p* < 0.05(*), *p* < 0.01(**), *p* < 0.001(***), *p* < 0.0001(****).

Three top pathways strongly associated with inflammation and immunity were selected from the functional enrichment analysis: Complement, Inflammatory Response, and IL6 JAK STAT3 Signaling. These three pathways contained a total of 39 genes. To identify diagnostic biomarkers, inflammation-related DEGs were first screened by univariate logistic regression (*p* < 0.05) (Figure 3A). Subsequently, two distinct machine learning algorithms were employed for feature selection: (1) Least absolute shrinkage and selection operator (LASSO) regression was performed using the R package glmnet with 10-fold cross-validation to determine the optimal penalty parameter (*λ*). Genes with non-zero coefficients at the *λ* min were retained (Figures 3B,C). (2) Random Forest algorithm was applied, and the top 10 genes ranked by mean decrease in Gini impurity were selected (Figure 4A). The intersection of genes from LASSO and Random Forest yielded eight candidate genes (Figure 4B). To build a parsimonious and stable diagnostic model, these eight genes were entered into a multivariate logistic regression model. A bidirectional stepwise selection procedure (with minimization of Akaike Information Criterion (AIC) as the goal) was then performed to refine the gene set. This final step resulted in a six-gene signature (DCBLD2, FZD5, GP1BA, MMP8, RNF144B, and SOCS1) (Figure 4C). The two excluded genes (ITGB3 and MARCO) were removed due to their non-significant independent contributions (*p* > 0.05) in the multivariate model.

### 3.4 Prediction model and nomogram construction

**Figure 5:**
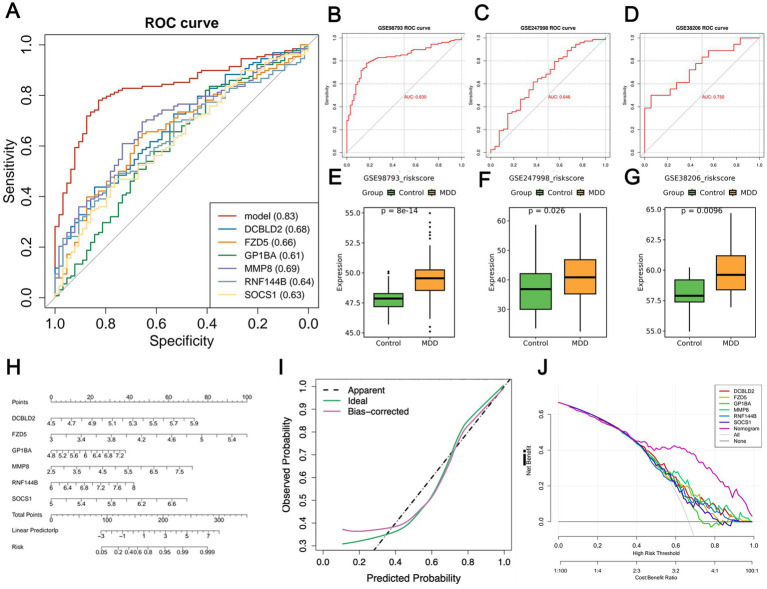
Construction, evaluation, and immune cell analysis of the MDD multi-gene model. **(A)** The ROC curve of the multi-gene model, with the highest AUC value of 0.83. **(B–D)** The ROC curves of the model in the training set (GSE98793) and validation cohorts (GSE247998, GSE38206). (**E–G**) Box plots indicating that the RS values of MDD samples are significantly higher than those of control samples across the three cohorts. (**H**) A nomogram predicting MDD risk based on six genes. (**I**) Calibration curve used to assess the diagnostic potential of the model. **(J)** DCA curve used to evaluate the practical effectiveness of the model.

A predictive model was constructed using six genes: DCBLD2, FZD5, GP1BA, MMP8, RNF144B, and SOCS1. The results indicated that the multi-gene model achieved the highest AUC value of 0.83 (Figure 5A), outperforming the other single-gene models. Furthermore, we utilized training and validation cohorts to assess the predictive capability of the established model. The AUC values of the ROC curve for the model in the training set (GSE98793) and validation cohorts (GSE247998, GSE38206) were 0.83, 0.646, and 0.750 (Figures 5B–D), respectively, demonstrating the model’s reliable predictive performance in diagnosing MDD. The RS values of MDD samples were significantly higher than those of control samples (Figures 5E–G). A nomogram model was generated to predict the risk of MDD based on the six genes. Each predictive marker was projected upwards to the “points” value at the top of the nomogram to obtain a score ranging from 0 to 100, and the total score of the six points was then calculated to predict the probability of MDD risk. This nomogram model, which integrates the expression of multiple genes, assists physicians in visually assessing the risk of MDD in patients (Figure 5H). The calibration curve demonstrated a strong agreement between the predicted MDD risk and the actual risk (Figure 5I). Additionally, decision curve analysis (DCA) indicated that the central gene curve was positioned above the gray line, suggesting that using the nomogram to predict MDD risk offers significant net benefits (Figure 5J).

### 3.5 Immune infiltration analysis and prefrontal data

**Figure 6:**
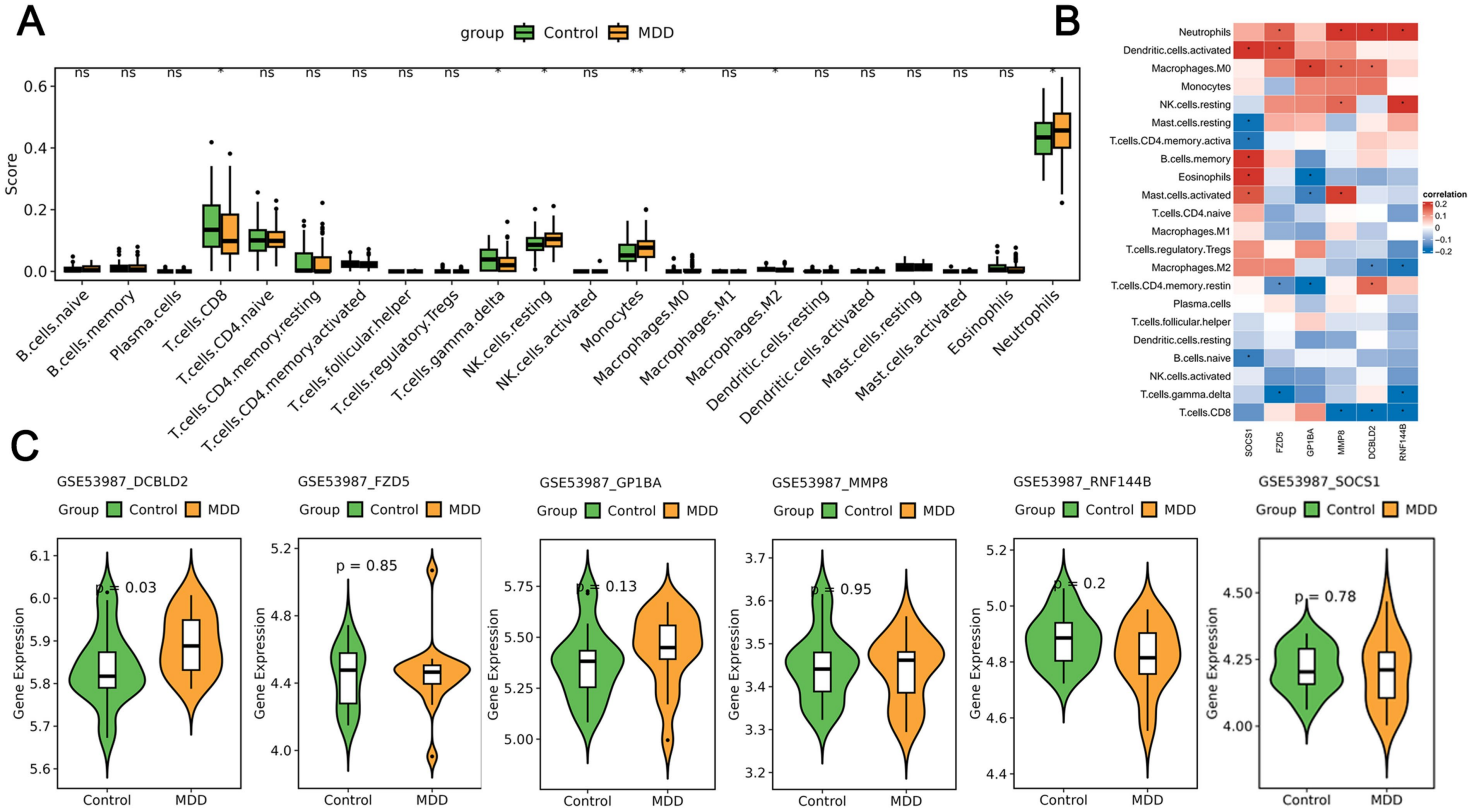
**(A)** Box plot showing the level of immune cell infiltration between the two groups in the training set (GSE98793). **(B)** Heatmap displaying the correlation between diagnostic genes and immune cell types. **(C)** Validation of the expression levels of six candidate genes in prefrontal samples of MDD and control groups from GSE53987.

To observe the immune status of the control group and the MDD group, we utilized CIBERSORT to compare the levels of immune cell infiltration between the two groups. The results indicated significant differences in the infiltration levels of seven immune cell types between the two groups. In brief, compared to the control samples, the MDD samples exhibited lower levels of CD8T, gamma deltaT, and M2 macrophages, but higher levels of M0 macrophages, resting NK cells, monocytes, and neutrophils (Figure 6A). This immune profile suggests a state of innate immune activation coupled with impaired adaptive immunity in MDD. Subsequently, we analyzed the correlation between seven diagnostic genes and immune cell types. The results showed that the expression of four genes, DCBLD2, FZD5, MMP8, and RNF144B, had a significant positive correlation with neutrophils, while the expression of three genes, DCBLD2, MMP8, and RNF144B, had a significant negative correlation with CD8T cells (Figure 6B). To reinforce our immune infiltration findings, we employed ESTIMATE and ssGSEA algorithms. ESTIMATE analysis revealed a significantly decreased stromal score in MDD samples (*p* = 0.026) (Supplementary Figure S1). ssGSEA further confirmed an immune imbalance, showing increased scores for activated CD8 + T cells and neutrophils, but decreased scores for central memory CD4 + T cells and regulatory T cells in MDD (Supplementary Figure S2). These results, consistent with the CIBERSORT analysis, collectively underscore a complex immune dysregulation state in MDD. Based on the GSE53987 dataset, we analyzed the expression of six genes: DCBLD2, FZD5, GP1BA, MMP8, RNF144B, and SOCS1 in the prefrontal samples of MDD and Control, finding that DCBLD2 was significantly overexpressed in MDD samples (Figure 6C).

### 3.6 Single-cell transcriptomic analysis of human post-mortem prefrontal cortex

**Figure 7:**
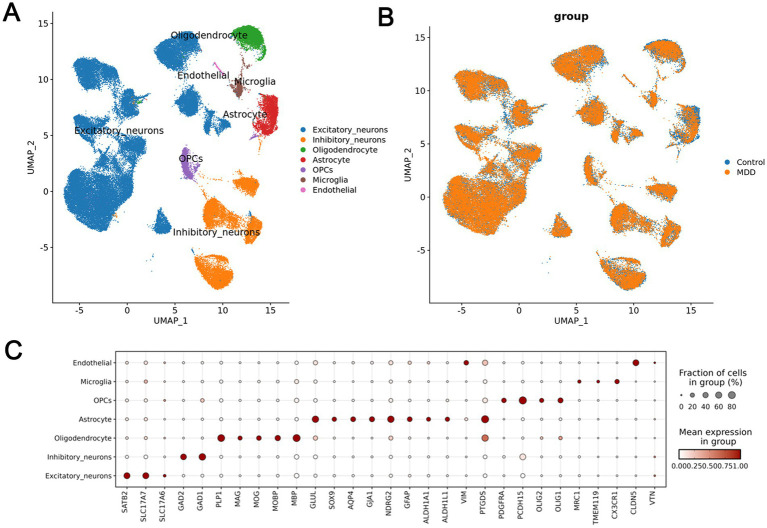
Single-cell transcriptomic analysis of human post-mortem dorsolateral prefrontal cortex. **(A)** The UMAP analysis plot displays the distribution of seven cell populations, including excitatory neurons, inhibitory neurons, oligodendrocytes, astrocytes, oligodendrocyte precursor cells, microglia, and endothelial cells. **(B)** The analysis shows the distribution differences between the MDD group (orange) and the control group (blue) across different cell types. **(C)** The expression levels of different genes in various cell types of the MDD group and the control group are presented, including the expression ratios and means within each group.

**Figure 8:**
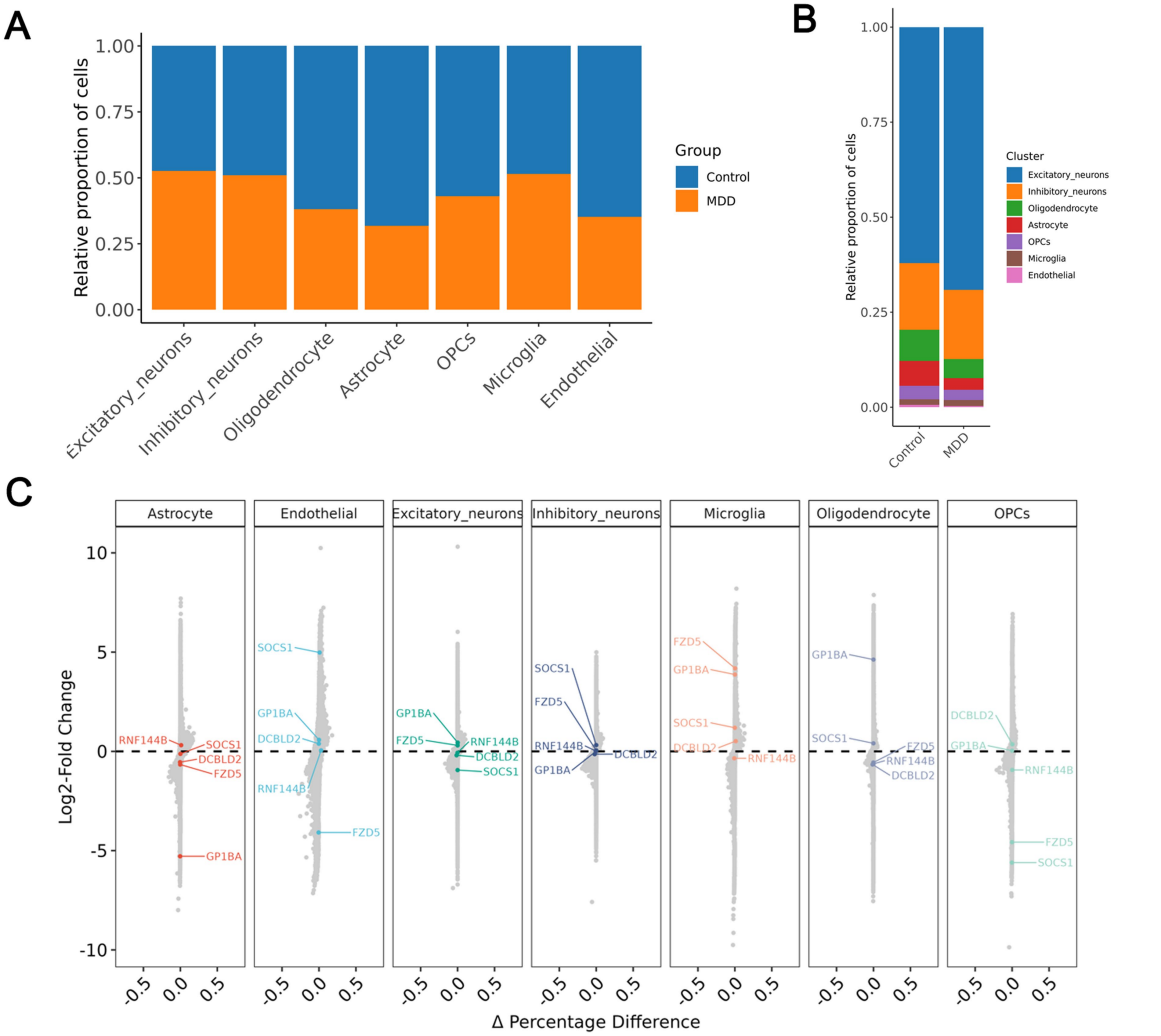
DCBLD2 expression in human post-mortem prefrontal cortex cell types. **(A)** A comparison of the relative proportions of MDD and control groups across each cell type. **(B)** Differences in the composition of cell distribution between the MDD and control groups within the cell populations. **(C)** The log2 fold changes and percentage differences of six candidate genes across different cell types.

We analyzed single-cell sequencing data derived from publicly available human post-mortem dorsolateral prefrontal cortex (BA9) tissues of subjects with Major Depressive Disorder (MDD) and control subjects (GSE144136). After quality control, a total of seven cell types were identified: Excitatory neurons, Inhibitory neurons, Oligodendrocytes, Astrocytes, Oligodendrocyte Progenitor Cells (OPCs), Microglia, and Endothelial cells (Figures 7A,B). We examined the expression levels of six genes, namely DCBLD2, FZD5, GP1BA, MMP8, RNF144B, and SOCS1, across various cell types in MDD and control post-mortem samples, and found that DCBLD2 was highly expressed in endothelial cells, Microglia, and OPCs in MDD (Figure 7C). Notably, the significant enrichment of DCBLD2 in these specific cell types of human MDD patients suggests its potential role in neurovascular unit dysfunction and neuroinflammation (Figures 8A–C).

### 3.7 Validation of the six-gene signature in human peripheral blood

**Figure 9:**
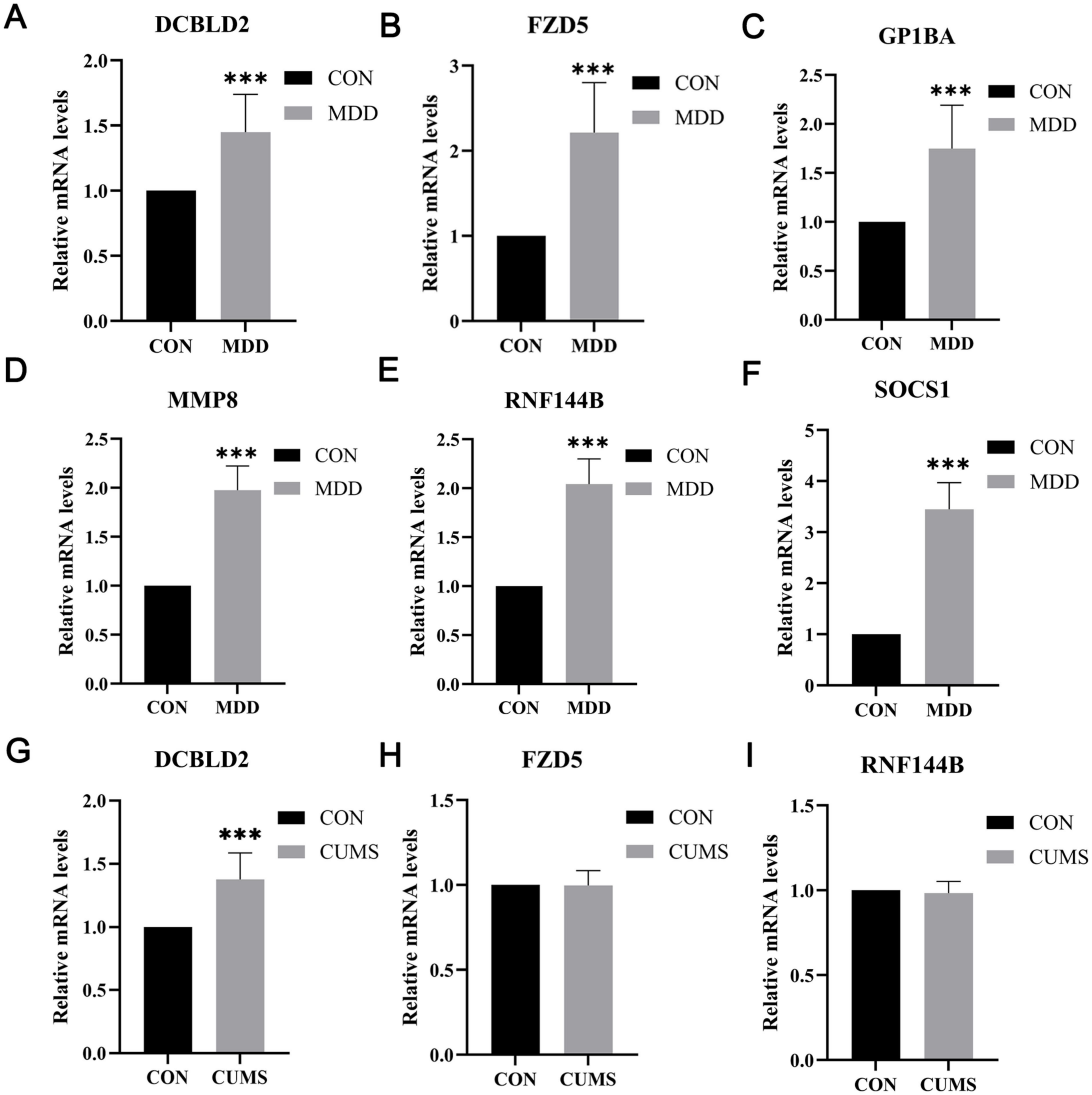
**(A)** Relative mRNA expression levels of DCBLD2 in the MDD and control groups. **(B)** Relative mRNA expression levels of FZD5 in the MDD and control groups. **(C)** Relative mRNA expression levels of GP1BA in the MDD and control groups. **(D)** Relative mRNA expression levels of MMP8 in the MDD and control groups. **(E)** Relative mRNA expression levels of RNF144B in the MDD and control groups. **(F)** Relative mRNA expression levels of SOCS1 in the MDD and control groups. **(G)** Relative mRNA expression levels of DCBLD2 in the CUMS and control groups. **(H)** Relative mRNA expression levels of FZD5 in the CUMS and control groups. **(I)** Relative mRNA expression levels of RNF144B in the CUMS and control groups.

To evaluate the clinical diagnostic potential of the identified biomarkers, we performed RT-qPCR analysis using peripheral blood samples from an independent human clinical cohort. The results confirmed that the mRNA levels of all six candidate genes (DCBLD2, FZD5, GP1BA, MMP8, RNF144B, and SOCS1) were significantly upregulated in the peripheral blood of human MDD patients compared to healthy controls (Figures 9A–F). These findings demonstrate that the six-gene diagnostic panel is a robust indicator of MDD in human clinical samples.

### 3.8 Experimental validation of candidate genes in a CUMS rat model

**Figure 10:**
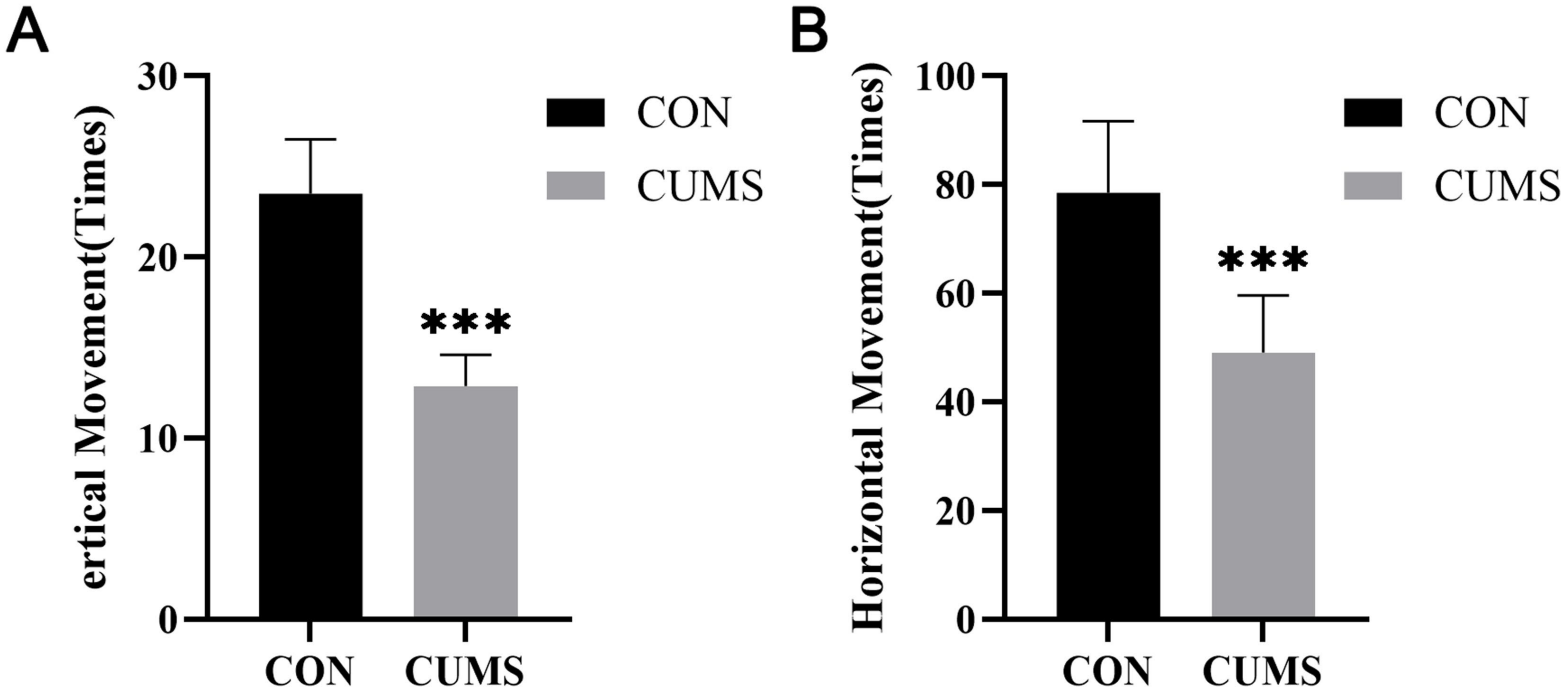
Open field test **(A)** vertical movement counts were reduced in the CUMS group compared with controls. **(B)** Horizontal movement counts were reduced in the CUMS group compared with controls.

To further explore the central expression of these genes in a controlled pathological state, we established a Chronic Unpredictable Mild Stress (CUMS) rat model. Behavioral assessments were conducted to confirm the validity of the model. Before stress intervention, no significant differences were observed in baseline behavior between the control and model groups. However, after 28 days of CUMS, the model group exhibited significant reductions in both vertical and horizontal movements in the open field test compared to the control group (Figures 10A,B), indicating the successful induction of depressive-like behaviors. Subsequently, we analyzed gene expression in the prefrontal cortex of the rats. While DCBLD2 expression was significantly elevated in the rat PFC (Figure 9G), the expression levels of RNF144B and FZD5 did not show significant differences (Figures 9H, I). These results highlight DCBLD2 as a core gene consistently dysregulated across both human and rodent models, reinforcing its significance in MDD pathophysiology.

## 4 Discussion

Major depressive disorder is a severe illness that affects millions of people worldwide, placing a heavy burden on families and communities. Therefore, there is an urgent need to develop reliable clinical detection methods. This study aims to screen for candidate genes associated with MDD and explore their potential diagnostic value. Our enrichment analyses paint a nuanced picture of immune dysregulation in MDD. While up-regulated genes highlight innate immune and inflammatory pathway activation (e.g., TLR signaling, complement), the down-regulated gene set reveals a concurrent suppression of fundamental cellular processes and adaptive immune signaling pathways (e.g., JAK–STAT, T cell receptor). This duality suggests that MDD may involve not merely a state of generalized inflammation, but a disrupted balance between pro-inflammatory drives and homeostatic/regulatory immune functions, potentially contributing to both systemic symptomatology and impaired neural resilience. In this research, we constructed a diagnostic model based on six MDD-related genes. Validation results indicate that this model has good diagnostic performance and is closely related to the level of immune cell infiltration.

Our study employed a two-phase strategy. First, we used Mendelian randomization analysis to provide genetic support for a significant association between inflammatory dysregulation and MDD, thereby establishing the “inflammatory-immune” axis as the central investigative direction for this study. The study first identified two cytokines associated with MDD through MR, finding that elevated levels of IL-9 increase the risk of MDD, while increased levels of MIP-1β decrease the risk. IL-9 is a cytokine secreted by Th9 cells, which activates the cross-phosphorylation of JAK1 and JAK3 upon binding to its receptor, leading to the activation of signal transducer and activator of transcription factors 1, 3, and 5 (Renauld, 2001). This signaling pathway plays a crucial role in immune regulation and is closely related to the pathophysiology of depressive disorders. Studies indicate that the role of IL-9 in depressive disorders is reflected in its expression within the central nervous system. For instance, post-mortem analyses of Brodmann area 10 in the brains of MDD patients revealed a significant upregulation of IL-9 gene expression (Shelton et al., 2011). Brodmann area 10 is a key brain region involved in reward behavior and emotional regulation, suggesting that IL-9 may influence the neurobiological mechanisms underlying depressive disorders. Additionally, clinical studies have found that elevated levels of IL-9 are associated with depressive and anxiety symptoms during mid-pregnancy in women, further supporting the potential role of IL-9 in mood disorders (Karlsson et al., 2017). In contrast, MIP-1*β* is a chemokine that plays a significant role in immune regulation and inflammatory responses. Leighton et al., 2018 observed that a decrease in MIP-1*β* levels in healthy individuals is associated with an increased risk of depressive disorders (Leighton et al., 2018), which further supports the potential role of MIP-1β in depression. These findings suggest that inflammation-related pathways play an important role in the pathophysiology of MDD, providing a logical starting point and rationale for our subsequent investigation into the downstream transcriptional events associated with inflammatory dysregulation in MDD at the transcriptomic level. Building on this, we proceeded to identify differentially expressed genes strongly associated with inflammation at the transcriptomic level. We focused on selecting candidate biomarkers from the upregulated differentially expressed genes for diagnostic model construction. The enrichment results of downregulated genes, in contrast, suggested a potentially concomitant state of immune regulatory suppression in MDD, providing a complementary perspective for understanding the complexity of the disorder. Finally, machine learning was utilized to determine six diagnostic genes: DCBLD2, FZD5, GP1BA, MMP8, RNF144B, and SOCS1. A nomogram prediction model was constructed based on these six diagnostic genes, and the accuracy of the prediction model was evaluated using the ROC curve from an external validation dataset.

Among the identified candidates, DCBLD2 emerges as a particularly compelling lead. Its consistent upregulation across peripheral blood and the prefrontal cortex, coupled with its specific enrichment in microglia and endothelial cells at single-cell resolution, positions it at the nexus of neurovascular and neuroimmune interactions. Although direct studies in MDD are lacking, its known roles in endothelial activation and immune cell recruitment in other pathologies support the hypothesis that DCBLD2 may facilitate blood–brain barrier dysfunction and amplify neuroinflammatory cascades in MDD, warranting prioritized functional investigation.

MMP8 (Matrix Metallopeptidase 8) is a matrix metalloproteinase (Juurikka et al., 2019), a member of the MMPs family, primarily secreted by neutrophils and macrophages, and is involved in the degradation and remodeling of the extracellular matrix (ECM). Studies have shown that the expression of MMP8 is increased in the serum of patients with MDD as well as in mouse models of psychosocial stress (Cathomas et al., 2024). Moreover, stress can induce peripheral immune cells to release MMP8, which alters the neuronal microenvironment by degrading basement membrane components such as laminin, closely related to the decreased synaptic plasticity in the prefrontal cortex of MDD patients. The study also indicates that reducing the expression of MMP8 can prevent stress-induced social avoidance behavior and changes in the neurophysiology and extracellular space of the nucleus accumbens (NAc).

SOCS1 (Suppressor of Cytokine Signaling 1) is a “brake” on cytokine signals. Several studies report lower SOCS1 in people with MDD. In PBMCs from MDD patients, SOCS1 mRNA is lower than in healthy controls (Kobayashi et al., 2022) (Lin and Huang, 2021). In CD14^+^ monocytes from MDD patients, SOCS1 is also reduced (Hung, 2018). These results suggest that the decreased expression of SOCS1 may be closely related to the pathological mechanisms of MDD. SOCS1 keeps immune and inflammatory balance by blocking the JAK–STAT, NF-κB, and MAPK pathways. In patients with MDD, low SOCS1 may allow excessive inflammation. Lower SOCS1 is linked to higher TNF-*α*, IL-1*β*, and IL-6 (Sun et al., 2022) (Kobayashi et al., 2022). Studies have also found that Some antidepressants raise SOCS1 mRNA in patient monocytes and restore control of TLR-driven inflammation. This action may partly explain improvements in mood and inflammation (Lin and Huang, 2021) (Hung, 2018). Furthermore, in the neuroinflammatory model of MDD, the reduced expression of SOCS1 is closely related to neuronal damage. MiR-345-5p activates microglia and induces apoptosis of hippocampal neurons by inhibiting the expression of SOCS1 (Liu et al., 2020) (Sun et al., 2019). Overexpression of SOCS1 can reverse this pro-inflammatory effect, indicating the important role of SOCS1 in protecting neurons from damage. Our study consistently identified SOCS1 as a diagnostic gene, and its downregulation in our model aligns with this pro-inflammatory hypothesis, potentially serving as a critical node in MDD-related immune dysfunction.

FZD5 (Frizzled-5) is a key receptor in the classical Wnt signaling pathway, which is widely involved in regulating neuronal development, axon guidance, and synaptic plasticity, playing an important role in brain structure formation and emotional regulation(Mulligan and Cheyette, 2017). Previous studies have shown that Wnt signaling is crucial for cell fate determination, neuronal migration, and synaptic connections during embryonic development. When this pathway is disrupted, disease risk rises, and researchers link it to cancer and depression (Anastas and Moon, 2013). In both humans and animal models, impaired function of the Wnt/*β*-catenin pathway can induce hippocampal shrinkage (Wang et al., 2022). Significant downregulation of FZD5 expression has been observed in mouse models subjected to chronic stress, accompanied by reduced hippocampal volume, neuronal damage, and the emergence of depressive-like behavior (Yan et al., 2023; Ye et al., 2023).

GP1BA is a platelet glycoprotein that is an important component of the coagulation response. Elevated levels of GP1BA expression in the peripheral blood of patients with MDD indicate excessive platelet activation, which may amplify systemic inflammation through serotonin depletion and cytokine release (such as transforming growth factor-β and platelet factor) (R et al., 2022) (Neubauer et al., 2013). This is consistent with the complement and coagulation cascade pathways enriched in our analysis related to major depressive disorder.

RNF144B is an E3 ubiquitin ligase involved in protein degradation and apoptosis. Previous studies have linked it to lung adenocarcinoma and ovarian cancer, primarily through regulation of the p53 axis, and have associated its abnormalities with spermatogonial stem cell dysfunction and azoospermia. In our multivariate regression analysis, RNF144B expression showed a marginally significant negative association with MDD (*p* = 0.076), suggesting its downregulation may be related to the disease. However, this gene did not show differential expression in the central nervous system in CUMS animal models. Therefore, its specific role in human emotion-related circuits (e.g., hippocampus and prefrontal cortex), and whether it promotes neuroinflammation by impairing clearance of damaged proteins, increasing oxidative stress, or disrupting mitochondrial-apoptosis balance, remains to be experimentally verified.

DCBLD2 (Discoidin, CUB and LCCL Domain Containing 2) is a transmembrane protein-coding gene located on chromosome 3 (Schmoker et al., 2019), belonging to the neurofibrillary protein family, with core functions involving vascular remodeling and endothelial homeostasis regulation. Although there is currently a lack of direct studies on the function of DCBLD2 in the context of major depressive disorder, previous evidence suggests that DCBLD2 can regulate cell migration, adhesion, and inflammatory signaling pathways. For instance, in atherosclerosis models (Nie et al., 2013), DCBLD2 overexpression was shown to promote endothelial cell activation and monocyte recruitment; in glioblastoma studies (Feng et al., 2025), DCBLD2 was found to enhance the infiltration of tumor-associated macrophages and immune suppression (Schmoker et al., 2017). These results support the hypothesis of this study that DCBLD2 may enhance the recruitment and activation of immune cells in the central nervous system, thereby amplifying neuroinflammatory cascades. The results of this study suggest that DCBLD2 may support the exacerbation of neuroinflammatory cascades by enhancing the recruitment and activation of immune cells in the central nervous system. This research further supports the notion that abnormal expression of DCBLD2 may promote the onset and progression of MDD. Additionally, the critical role of DCBLD2 in hypertension and angiogenesis disorders indicates that it could serve as a shared molecular target for comorbidities such as post-stroke depression. Our findings, which demonstrate a significant upregulation of DCBLD2 in prefrontal cortex of MDD subjects and its specific enrichment in microglia and endothelial cells at single-cell resolution, provide the first direct evidence supporting its role in MDD pathophysiology, likely through mediating neurovascular and neuroinflammatory processes.

Given the distinct biological profiles of these genes, our experimental validation aimed to dissect their potential roles in the peripheral versus central nervous systems. Our analysis revealed that in the human post-mortem pre-frontal cortex data (GSE53987), only DCBLD2 showed significant upregulation in MDD patients (Figure 6C). Coupled with its specific enrichment in microglia and endothelial cells of MDD patients at the single-cell level (Figures 7, 8), we designated DCBLD2 as the core target for validation, hypothesizing its role in linking peripheral immunity to central neuroinflammation. Although the expression changes of FZD5 and RNF144B in GSE53987 did not reach statistical significance, we nonetheless examined them in the rat prefrontal cortex. This decision was based on two rationales: First, the processes regulated by FZD5 (Wnt signaling) and RNF144B (ubiquitin-proteasome system)—neuroplasticity and protein homeostasis—are well-established key aspects of depressive pathophysiology, complementing the vascular and immune regulatory pathways associated with DCBLD2. Second, unlike GP1BA, MMP8, and SOCS1, which are primarily expressed and functional in peripheral immune cells (e.g., platelets, neutrophils), FZD5 and RNF144B have documented basal expression in brain tissue. Therefore, assessing their alterations in the central chronic stress environment contributes to a more comprehensive understanding of the biological basis of this multi-gene diagnostic signature. Ultimately, animal experimental results demonstrated that in the prefrontal cortex of CUMS model rats, only DCBLD2 expression was significantly upregulated, while no significant changes were observed for FZD5 or RNF144B (Figures 9G–I). This aligns with our human brain transcriptomic data, suggesting that the six-gene signature may have different dominant players in the central versus peripheral compartments: The alteration of DCBLD2 appears more specific and prominent within the brain, potentially serving as a key nexus mediating the impact of peripheral immune abnormalities on prefrontal cortex function. In contrast, the diagnostic value of the other genes, particularly GP1BA, MMP8, and SOCS1, may stem more predominantly from their reflection of peripheral immune status.

Major Depressive Disorder exhibits a strong link to immune system dysregulation, marked by the abnormal presence of immune cells and changes in immune reactions. This phenomena encompasses a reduction in the phagocytic capabilities of leukocytes, lymphocytes, and neutrophils circulating in the blood, while simultaneously showing an increase in the phagocytic activity of monocytes. In addition to these shifts in immune cell demographics, there are various variations in inflammatory immune mediators, including TNF-*α*, CRP, IL-6, SAA, and INF-*γ*. The findings from this study indicate that individuals with MDD often reside in a pro-inflammatory setting primarily driven by innate immunity, with notably heightened levels of intrinsic immune cells (such as M0 macrophages, monocytes, resting NK cells, and neutrophils), contrasted with reduced levels of adaptive immune cells (including CD8 T cells and γδ T cells). This discrepancy in immune function not only signifies an excessive activation of the innate immune response but may also contribute to the persistence of chronic inflammation. Chronic stress appears to exacerbate the dysregulation of the neuro-immune axis through the prolonged activation of the immune system. Concurrently, the elevated levels of these immune mediators may intensify depressive symptoms during the ongoing inflammatory response. Our immune infiltration analysis, revealing an innate-adaptive immune imbalance, complements the gene expression findings. The positive correlation between DCBLD2/MMP8 and neutrophils, and their negative correlation with CD8T cells, suggests a potential mechanism whereby these molecules contribute to the recruitment of innate immune cells and suppression of adaptive immunity, creating a chronic inflammatory milieu conducive to MDD.

This pervasive immune imbalance signifies not only an over activation of the innate immune response but also potentially underpins the persistence of a chronic inflammatory state. Chronic stress is posited to foster dysregulation of the neuro-immune axis by inducing a prolonged state of immune system activation. Concurrently, the elevated expression of key inflammatory mediators likely exacerbates depressive symptomatology throughout the sustained inflammatory process.

Microglia, the resident immune sentinels of the central nervous system, constitute the primary defense against pathogenic invasion. Evidence suggests that chronic stress propels the production of inflammatory monocytes, facilitating their active recruitment to the prefrontal cortex and subsequently amplifying neuroinflammatory responses. Under pathological conditions—including trauma, infection, and psychological stress—microglia rapidly respond to stimulus signals. Once activated, they secrete a plethora of pro-inflammatory factors (e.g., IL-1β, TNF-α, IL-6), precipitating synaptic loss and neurological dysfunction, which ultimately manifest as depressive-like behaviors. Leveraging public post-mortem database mining, this study identified that DCBLD2 is significantly upregulated in the prefrontal cortex of Major Depressive Disorder (MDD) patients. Its elevated expression was predominantly localized to microglia, endothelial cells, and oligodendrocyte precursor cells (OPCs).

In summary, this integrative study synthesizes multi-level evidence across molecular, cellular, and behavioral phenotypes to propose a plausible mechanistic model: chronic systemic inflammation disrupts blood–brain barrier integrity, facilitating the infiltration of monocytes/macrophages exhibiting high DCBLD2 expression into brain tissue. These cells may differentiate into microglia-like cells that regulate critical downstream signaling pathways, thereby exacerbating central neuroinflammation, impairing neural plasticity, and ultimately triggering depressive-like behavior.

This research possesses several limitations: (1) Limited sample size: The animal model investigations incorporated a relatively modest sample size, which may introduce statistical bias or limit generalizability. Future studies should seek to validate model stability and applicability using larger-scale and multi-center cohorts. (2) Lack of direct functional validation of molecular mechanisms: Although bio informatic inference, single-cell transcriptomic profiling, and behavioral validation in models support the potential involvement of DCBLD2, MMP8, SOCS1, and FZD5 in MDD pathogenesis, direct *in vivo* functional experiments—such as cell-specific gene knockout or overexpression—are required to establish causality. Subsequent research could employ microglia-specific conditional knockout or overexpression models to elucidate the functional roles of these genes. (3) The dynamic process of peripheral-central immune crosstalk remains uncharacterized: While this study proposes a hypothesis wherein peripheral inflammation mediates microglial activation via blood–brain barrier disruption, it does not directly trace monocyte migration or microglial phenotypic transformation. Future work could integrate single-cell spatial transcriptomics or in vivo cell lineage tracing techniques to delineate dynamic changes in the peripheral-central immune axis throughout MDD progression. (4) Limitations in Data Availability: The sample information available in the public databases used (e.g., GSE98793, GSE247998) is limited. Case and control groups were balanced regarding obtainable variables such as sex and age. However, due to the absence of sample-level detailed comorbidity data, we were unable to adjust for such potential confounders. Therefore, while the identified biomarkers are associated with MDD, their specificity across different clinical subgroups warrants further validation in future studies.

## 5 Conclusion

In conclusion, this study identifies a panel of six candidate biomarkers—DCBLD2, FZD5, GP1BA, MMP8, RNF144B, and SOCS1—for Major Depressive Disorder. Among these, DCBLD2 was further experimentally validated across systems—in human peripheral blood, public human post-mortem prefrontal cortex datasets, and a rat model—demonstrating consistent dysregulation linked to MDD and representing a particularly promising candidate target.

## Data Availability

The datasets presented in this study can be found in online repositories. The names of the repository/repositories and accession number(s) can be found in the article/Supplementary material.

## Supplementary material

SUPPLEMENTARY FIGURE S1 Comparison of the stromal score assessed by the ESTIMATE algorithm.

SUPPLEMENTARY FIGURE S2 Immune cell infiltration profile assessed by the ssGSEA algorithm.
