# Supplementary material for: Integrative analysis identifies DCBLD2 and immune-related biomarkers for major depressive disorder: evidence from human peripheral blood, post-mortem brain, and rat models
Source: Front Hum Neurosci. 2026 Apr 30;20:1770103. doi: 10.3389/fnhum.2026.1770103 (PMC13171845; doi:10.3389/fnhum.2026.1770103)
Supplement: SUPPLEMENTARY FIGURE S1 — Comparison of the stromal score assessed by the ESTIMATE algorithm. [file Supplementary_file_1.zip › Supplementary Material/Supplementary File_1.DOCX]

Supplementary Material

Materials and methods

# Data Collection

The GWAS data for MDD were obtained from the OpenGWAS database (https://gwas.mrcieu.ac.uk), which includes 135,458 cases and 344,901 control samples. The study utilized the following datasets GSE98793 (training set), GSE247998 (validation set 1: peripheral blood), GSE38206 (validation set 2: PBMCs), GSE53987 (prefrontal cortex bulk RNA-seq), GSE144136 (prefrontal cortex scRNA-seq).

# Mendelian Randomization Analysis

## Data Sources

The GWAS data for 41 inflammatory cytokines originate from a study that provided genomic variation associations with 41 cytokines and growth factors in 8,293 Finnish individuals (PMID: 27989323).

## Instrument Variable Selection

First, for each inflammatory cytokine, Single nucleotide polymorphisms (SNPs) were selected with a genome-wide significance threshold of p < 1 × 10^^-5^. Secondly, we clustered these SNPs using a threshold of R² > 0.1 and within 500 kb to eliminate linkage disequilibrium. Thirdly, we calculated the F-statistic for the instruments of each cytokine. An F-statistic threshold of > 10 was applied to select strong instruments for further MR analysis.

## Mendelian Randomization Analysis

The primary analytical method employed in the MR analysis is the Inverse Variance Weighted (IVW) method, combined with random effects, to generate heatmaps. Additionally, MR-Egger, weighted median, simple mode, and weighted mode were also used as auxiliary analysis methods.

## Sensitivity Analysis

The IVW Q test and MR-Egger test were used to check the heterogeneity of the MR analysis results, with a p-value greater than 0.05 indicating no heterogeneity. MR-Egger regression and Mendelian Randomization Pleiotropy RESidual Sum and Outlier (MR-PRESSO) were used to examine horizontal pleiotropy, ensuring that genetic variation is independently associated with exposure and outcome, with a p-value greater than 0.05 indicating no pleiotropy. MR-PRESSO was further applied to identify and exclude potential outliers, providing adjusted results. Leave-one-out analysis was utilized to assess whether causal associations might be driven by any single SNP.

# Differential Gene Analysis

The R package limma (v3.58.1) was used to analyze differentially expressed genes (DEGs) between normal control samples and MDD samples in the GSE98793 dataset, with screening criteria of |log2FC| > 0.5 & adjusted p-value (FDR) < 0.05, generating a volcano plot. GO (BP, CC, MF), KEGG, and Hallmark enrichment analyses were conducted using the R package clusterProfiler^[14]^, with pvalue < 0.05 indicating significant enrichment. The package visualized the expression patterns of DEGs, and the top 15 enriched pathways were plotted.

# Univariate Logistic Regression Analysis

To initially screen for inflammation-related DEGs with diagnostic potential, a univariate logistic regression analysis was performed using the rms package in R. Genes with a p-value < 0.05 were retained for further analysis.

# Model Construction

The Lasso and Random Forest (RF) algorithms implemented in R were used for further selection of DEGs associated with MDD. Subsequently, a multivariable logistic regression analysis was conducted using the R package RMS, and the best genes were selected as modeling genes. Risk scores (RS) were calculated based on the expression and coefficients of each diagnostic gene, leading to the construction of the diagnostic model:

P(target=1)=1/(1+exp(-(-47.86+2.72*DCBLD2+2*FZD5+0.76*GP1BA+

0.68*MMP8+1.1*RNF144B+2.01*SOCS1)))

# Nomogram Model Construction

To predict the incidence of MDD, a diagnostic nomogram model was established using the rms package. Calibration curves and Decision Curve Analysis (DCA) were employed to evaluate the predictive capability and practical application value of the model.

# Immune Infiltration

CIBERSORT, a computational tool for assessing the proportions of different cell types in gene expression data, was utilized in the training set to evaluate the distribution of immune cells in samples of MDD.

# Single-Cell RNA Sequencing Data Analysis

Single-cell data were analyzed using Seurat v4.1.1, filtering out cells with mitochondrial content exceeding 20%, hemoglobin content exceeding 5%, or gene expression of fewer than 200 or more than 8000 genes. Data normalization, cell clustering, and dimensionality reduction were performed using the Seurat package. The FindVariableFeatures function was used to select 2000 highly variable genes from the corrected expression matrix, followed by principal component analysis (PCA) using the RunPCA function, retaining the top 20 principal components for further analysis. Batch effects were corrected using the RunHarmony function from the R package harmony. Cell clustering was performed with the FindClusters function (resolution 0.6), and non-linear dimensionality reduction was conducted using the RunUMAP function. Cell clustering was annotated based on the CellMarker database and manually collected cell-specific markers.

# Animals and housing conditions

Sixteen male Sprague-Dawley (SD) rats (8-9 weeks old, weighing 200-220 g) were purchased from Spefu (Beijing) Biotechnology Co., LTD., with the production batch number N0110324231100438102, the Animal Experiment Center of Hunan University of Chinese Medicine. All animals were housed under a 12/12 h light/dark cycle in a controlled environment (temperature: 22 ± 2°C, humidity: 55 ± 10%) with ad libitum access to food and water. All efforts were made to minimize the pain and numbers of the animals used in the experiments. Use of Laboratory Animals and were approved by the Ethics Committee of Hunan Second People's Hospital (Approval No: 2022045).

# Study approval and subjects

The Ethics Committee of Hunan Second People's Hospital approved all materials and procedures. The clinical trial registration number is (2022045) .All participants or their legally authorized representatives provided written informed consent to participate in this study. Patients with MDD were recruited through inpatients of the Department of Hunan Second People's Hospital. Healthy control subjects were recruited through local community postings and media advertisements. Inclusion and exclusion criteria and behavioral measures are described in the Supplementary Information. All clinical samples were collected and experimented in accordance with WS/T 661-2020 Guidelines of venous blood specimen collection of the China Healthcare Commission and approved by the Ethics Committee of Hunan Second People's Hospital.

# CUMS model

Eight rats were randomly selected as the control group, while the remaining eight rats were subjected to the CUMS depression model. Various unexpected stressors were applied to the rats, including: (1) placing the rats in an inclined cage for 17 hours; (2) restricting the drinking time for the rats, with a 17-hour water deprivation; (3) fasting and water deprivation for 20 hours; (4) exposure to continuous light for 17 hours; (5) behavioral restriction for a duration of 2 hours; (6) placing the rats in a wet cage for 21 hours; (7) administering a mild electric shock (30V) to the rats' paws for 5 seconds; (8) allowing the rats to swim in ice water at 4°C for 5 minutes, with a water depth of 15 cm; and clamping the rats' tails for 1 minute. Over a period of 28 days, one type of stressor was randomly selected each day, ensuring that the same stressor did not occur on consecutive days. The open field behavior assessment was appropriately modified: an open field experimental apparatus made of opaque material measuring 100 cm × 100 cm × 50 cm was used, with the bottom evenly divided into 25 equal-sized squares. The rats were placed in the central square, and the number of squares traversed by the rats within 5 minutes (horizontal movement counts) and the number of times the rats stood on their forelimbs (vertical movement counts) were observed.

# Quantitative real-time PCR (qRT-PCR) Detection

Peripheral blood mononuclear cells (PBMCs) were isolated from fresh people blood samples using Ficoll density gradient centrifugation within 2 hours of collection. Total RNA was extracted from the blood samples using RNA extraction reagents. For rat prefrontal cortex tissue, 30-50 mg samples were collected after euthanasia, ground in liquid nitrogen, and RNA was isolated using TriQuick reagent. The RNA extraction process was the same for both sample types. Reverse transcription was performed using the Evo M-MLV reverse transcription kit at 37°C for 15 minutes and 85°C for 5 seconds. Quantitative PCR was conducted with the PerfectStart® Green qPCR SuperMix system, and gene expression was analyzed using ABI QuantStudio 1 PCR instrument software.

# Inclusion and exclusion criteria

For individuals in the MDD patients group, subjects were required to meet the following criteria: Inclusion criteria: (1) Meet the diagnostic criteria for MDD using a Structured Clinical Interview by two neuropsychiatrists according to the Diagnostic Statistical Manual of Mental Disorder, Fifth Edition (DSM-V); (2) Assessment of the severity of depressive symptoms using the 17-item Hamilton Depression Rating Scale (HAMD-17). (3) The age range of the included MDD patients was generally between 18 and 65 years. (4) Drug naïve or drug free for longer than three weeks (includes antidepressants and dependence-producing drugs such as narcotics), and no history of substance abuse; Exclusion criteria for healthy controls included history of neuropsychiatric disorders, head injury, substance abuse (includes antidepressants and dependence-producing drugs such as narcotics), or unconsciousness.

# Statistical Analysis

All experimental data were analyzed statistically using SPSS 27 64-bit software. The Shapiro-Wilk test was used to assess the normality of the data. Continuous variables are expressed as mean ± standard error of the mean (mean ± SEM) or median (interquartile range). Categorical variables are presented as n (%). In behavioral tests and gene expression validation (qPCR), comparisons between the control group and the CUMS model group were performed using two-tailed independent samples t-tests. The significance level for all statistical tests was set at p < 0.05. Statistical significance is indicated by asterisks, with the following standards: p < 0.05 (*), p < 0.01 (**), p < 0.001 (***), p < 0.0001 (****).
