# Supplementary figures and images for: Integrative analysis identifies DCBLD2 and immune-related biomarkers for major depressive disorder: evidence from human peripheral blood, post-mortem brain, and rat models
Source: Front Hum Neurosci. 2026 Apr 30;20:1770103. doi: 10.3389/fnhum.2026.1770103 (PMC13171845; doi:10.3389/fnhum.2026.1770103)

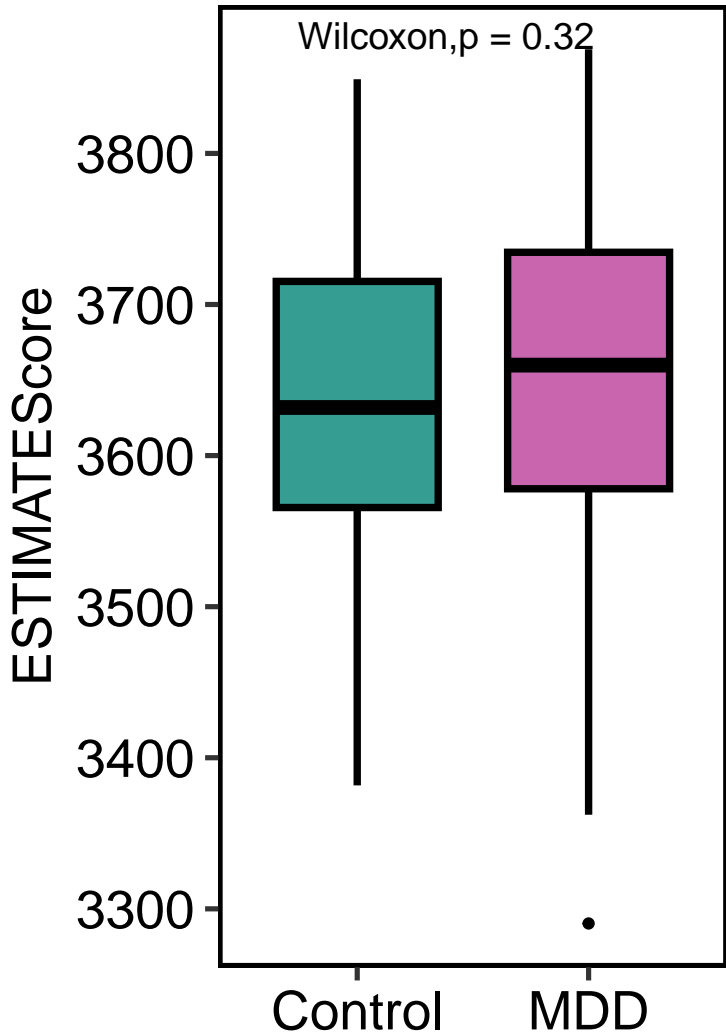

Supplement: SUPPLEMENTARY FIGURE S1 — Comparison of the stromal score assessed by the ESTIMATE algorithm. [file Supplementary_file_1.zip › Supplementary Material/Data Sheet 2.PDF]

group Control MDD

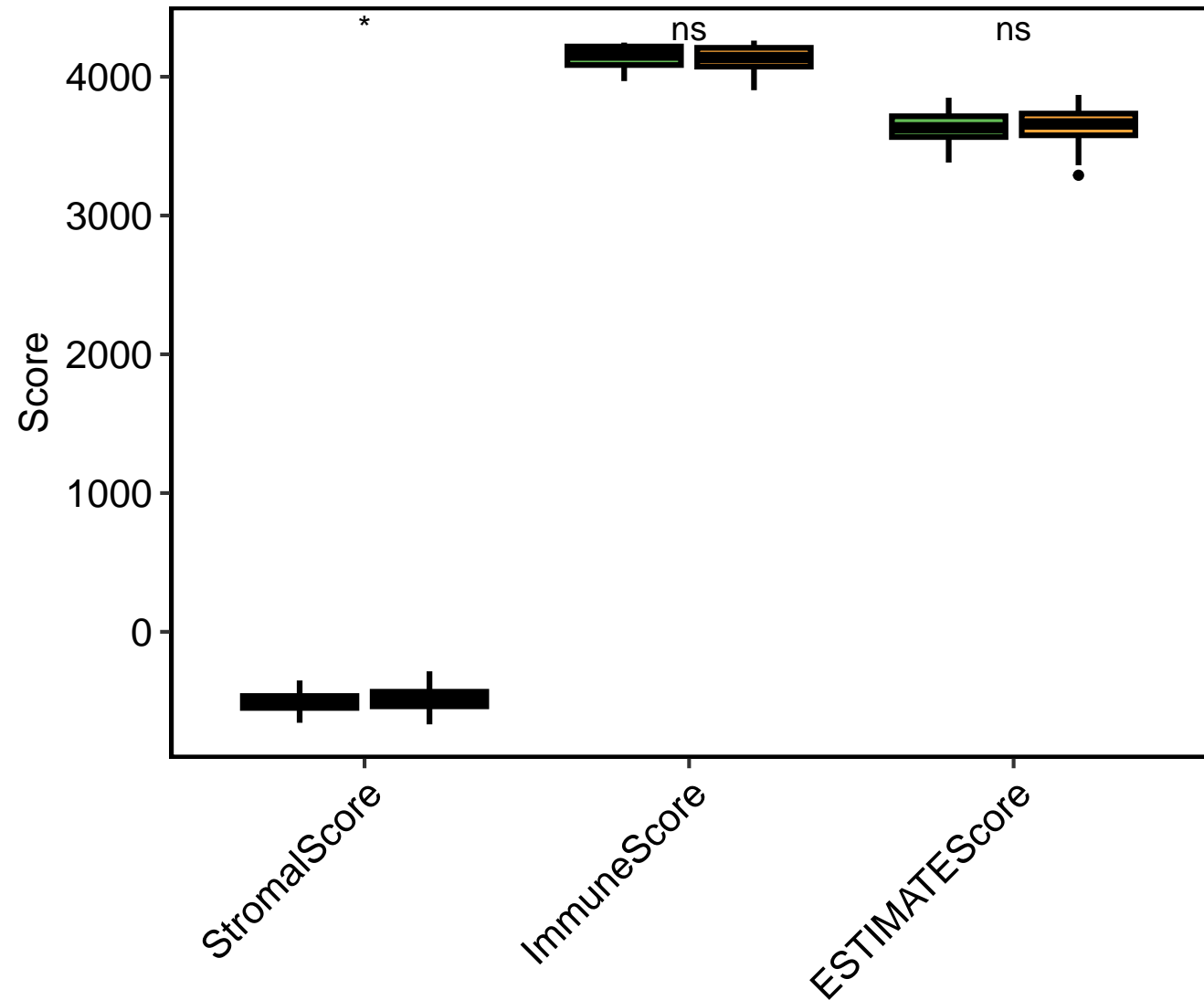

Supplement: SUPPLEMENTARY FIGURE S1 — Comparison of the stromal score assessed by the ESTIMATE algorithm. [file Supplementary_file_1.zip › Supplementary Material/Data Sheet 3.PDF]

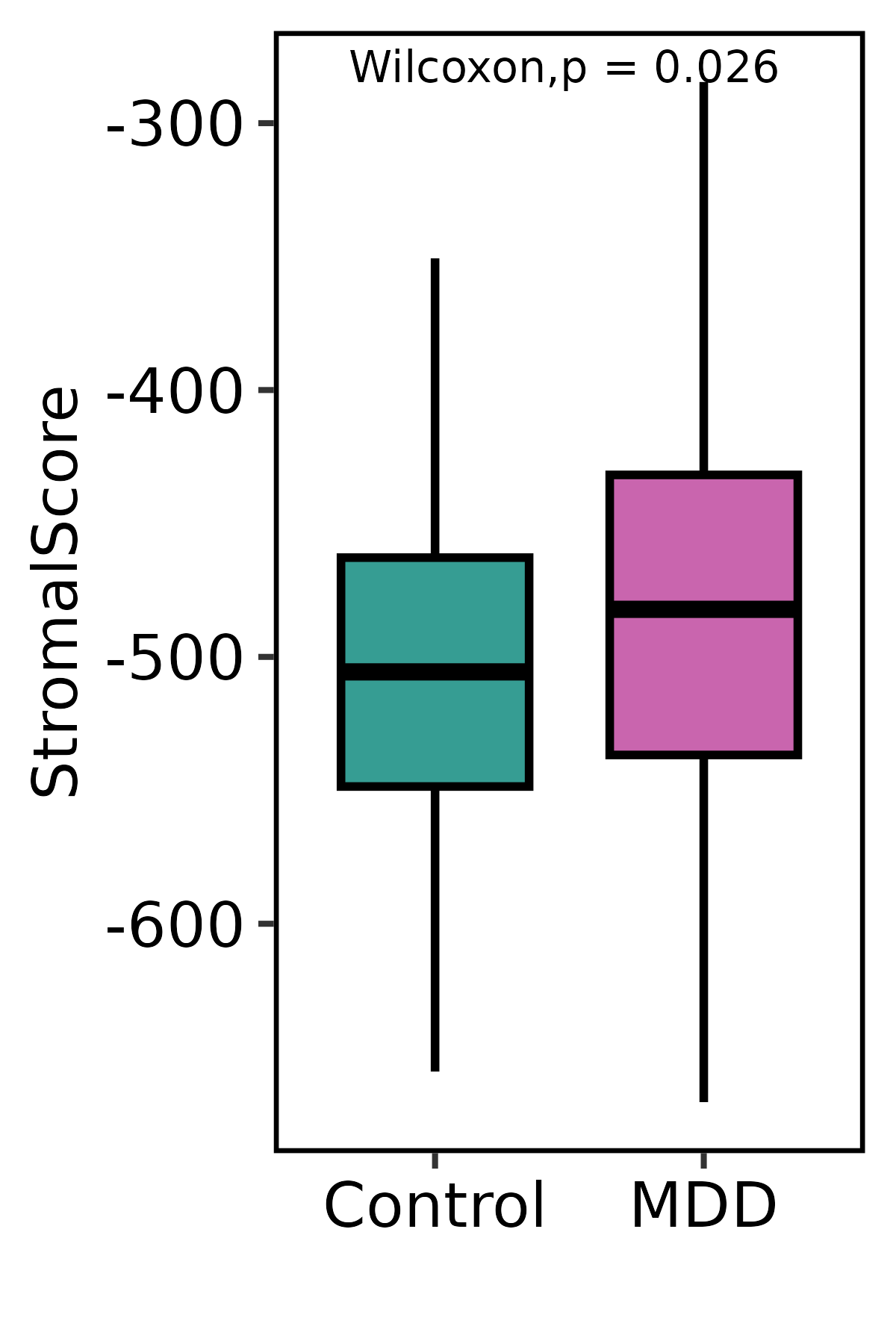

Supplement: SUPPLEMENTARY FIGURE S1 — Comparison of the stromal score assessed by the ESTIMATE algorithm. [file Supplementary_file_1.zip › Supplementary Material/Image 1.PNG]

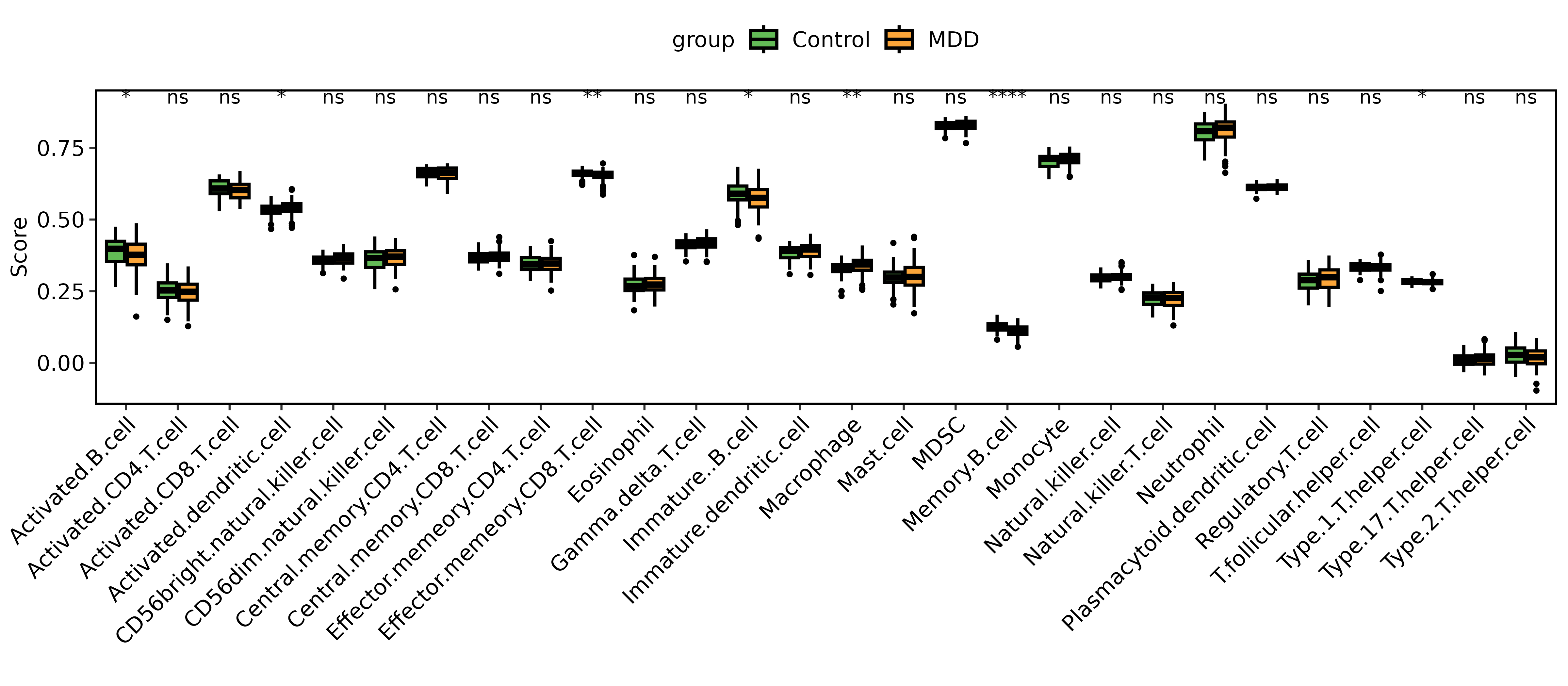

Supplement: SUPPLEMENTARY FIGURE S1 — Comparison of the stromal score assessed by the ESTIMATE algorithm. [file Supplementary_file_1.zip › Supplementary Material/Image 2.PNG]
